# Characterization and genomic analysis of a bensulfuron methyl-degrading endophytic bacterium *Proteus* sp. CD3 isolated from barnyard grass (*Echinochloa crus-galli*)

**DOI:** 10.3389/fmicb.2022.1032001

**Published:** 2022-10-24

**Authors:** Yanhui Wang, Xianyan Chen, Honghong Li, Yonglin Ma, Dongqiang Zeng, Liangwei Du, Decai Jin

**Affiliations:** ^1^Institute of Pesticide and Environmental Toxicology, Guangxi University, Nanning, China; ^2^Guangxi Key Laboratory of Biology for Crop Diseases and Insect Pests, Plant Protection Research Institute, Guangxi Academy of Agricultural Sciences, Nanning, China; ^3^College of Chemistry and Chemical Engineering, Guangxi University, Nanning, China; ^4^Key Laboratory of Environmental Biotechnology, Research Center for Eco-Environmental Sciences, Chinese Academy of Sciences, Beijing, China

**Keywords:** biodegradation, bensulfuron methyl, *Proteus* sp., extracellular enzyme, genomic analysis

## Abstract

Bensulfuron methyl (BSM) is a widely used sulfonylurea herbicide in agriculture. However, the large-scale BSM application causes severe environmental problems. Biodegradation is an important way to remove BSM residue. In this study, an endophytic bacterium strain CD3, newly isolated from barnyard grass (*Echinochloa crus-galli*), could effectively degrade BSM in mineral salt medium. The strain CD3 was identified as *Proteus* sp. based on the phenotypic features, physiological biochemical characteristics, and 16S rRNA gene sequence. The suitable conditions for BSM degradation by this strain were 20–40°C, pH 6–8, the initial concertation of 12.5–200 mg L^−1^ with 10 g L^−1^ glucose as additional carbon source. The endophyte was capable of degrading above 98% BSM within 7 d under the optimal degrading conditions. Furthermore, strain CD3 could also effectively degrade other sulfonylurea herbicides including nicosulfuron, halosulfuron methyl, pyrazosulfuron, and ethoxysulfuron. Extracellular enzyme played a critical role on the BSM degradation by strain CD3. Two degrading metabolites were detected and identified by using liquid chromatography–mass spectrometry (LC–MS). The biochemical degradation pathways of BSM by this endophyte were proposed. The genomic analysis of strain CD3 revealed the presence of putative hydrolase or esterase genes involved in BSM degradation, suggesting that a novel degradation enzyme for BSM was present in this BSM-degrading *Proteus* sp. CD3. The results of this research suggested that strain CD3 may have potential for using in the bioremediation of BSM-contaminated environment.

## Introduction

Bensulfuron methyl (BSM), 2-(4, 6-dimethoxypyrimidin-2-carbamoyl sulfamoyl)-o-toluic acid methyl ester, one of the most widely used sulfonylurea herbicides, is registered for the control of broadleaf and cyperaceous weeds in the paddy field (Si et al., 2004). As with other sulfonylurea herbicides, BSM is the inhibitor of acetolactate synthase (ALS), which is a key enzyme in the biosynthetic pathway of the branched amino acids in plants and microorganisms but not in animals. It exhibits the advantages with high herbicidal activity at low application rates and very low acute and chronic animal toxicity. However, the widespread and large-scale use of BSM over many years has raised increasing concerns about its residues in crops, soils and water, which significantly cause environment and health damage. In some countries, BSM has been frequently detected in rivers during crop seasons (Struger et al., 2011; Ismail et al., 2015). Previous studies showed that BSM caused toxicity to aquatic lives (Sabater et al., 2002; Yue et al., 2006; Chen et al., 2007), had phytotoxicity to rotation crops (Luo et al., 2008), and especially could result in some chronic diseases in human and animals (Zhu et al., 2005). Therefore, it is important to accelerate the decrease and elimination of BSM in the environment.

Microbial degradation was an important way to eliminate BSM residue in environment. Up to date, a lot of studies on the biodegradation of BSM by microorganism have been reported in literature. Some bacteria and fungi for the degradation of BSM were isolated from soil contaminated by sulphonylurea herbicides for several years (Zhu et al., 2005; Lü et al., 2008; Lin et al., 2010; Peng et al., 2012; Duc and Oanh, 2020; Hu et al., 2020) and activated sludge from the sludge tank of plant (Xiong et al., 2011). Relatively limited studies have been reported on the biodegradation of herbicides by using endophytic microbes that have mutualism effect on plants. It was demonstrated that endophytes played an important role to host plant resistant in polluted environment (Phillips et al., 2008; Afzal et al., 2014). In previous reports, many endophytic microbes had been isolated from different plants, which possessed pollutant-degrading activities (Yousaf et al., 2010; Oliveira et al., 2014). Several studies reported that endophytic bacteria could degrade herbicides including simazine (Ozawa et al., 2004), glyphosate herbicide (Kuklinsky-Sobral et al., 2005), quinclorac (Liu et al., 2014), diuron (Wang et al., 2017), and 4-chloro-2-methylphenoxyacetic acid (Wang et al., 2021), and so on. However, no study on the endophytic microbe to degrade BSM has been reported.

In this work, a BSM-degrading endophytic bacterium *Proteus* sp. CD3 was isolated from the root of barnyard grass collected from BSM-treated field. The main objectives of this work were to investigate the degradation characteristics of strain CD3, localize the degradation enzyme, identify the degradation metabolites of BSM, deduce the possible degradation pathway, and analyze the functional genes involved in BSM degradation. To our knowledge, it is the first report on the biodegradation of BSM by endophyte from barnyard grass.

## Materials and methods

### Chemicals and materials

Bensulfuron methyl (purity 98%) and other sulfonylurea herbicide standards (purity > 95%) were purchased from Dr. Ehrenstorfer GmbH (Augsburg, Germany). All solvents were HPLC grade (Sigma-Aldrich, United States) and other reagents were analytical grade (National Pharmaceutical Group Corporation, China). Unless otherwise stated, deionized water was used in all of the experiments.

The mineral salt medium (MSM) contained (g L^−1^): NH_4_NO_3_ 1.0, K_2_HPO_4_ 1.5, KH_2_PO_4_ 0.5, NaCl 1.0, MgSO_4_·7H_2_O 0.1, and FeSO_4_ 0.025. MSM amended with 10 g L^−1^ glucose (MMG) was also prepared. The composition of Luria–Bertani’s (LB) medium was as follows (g L^−1^): yeast extract 5, tryptone 10, NaCl 10. Solid media were prepared by adding 1.5% (w/v) agar into above-mentioned liquid media.

The barnyard grass in the vegetative growth phase before flowering was collected in July from a rice paddy filed in Wuming District of Nanning city, Guangxi, China (23°9′8.2476′′N, 108°11′54.9828′′E), where BSM has been applied for more than 10 years. The whole plants including roots, stems and leaves were placed in a labeled plastic bag, immediately transported to the laboratory where these plants were thoroughly washed in running tap water and a sonication step was employed to dislodge any soils and organic matters from the surface of plants.

### Isolation of BSM-degrading endophytic microbes

The surface of plants was sterilized by immersing in 75% (v/v) ethanol for 2 min, 0.1% mercuric chloride for 1 min, and then washed by sterile deionized water three times to clean the residues of sterilization agents. To verify the sterilization process, aliquots of the final sterile deionized water were spread onto LB agar plate and incubated at 37°C. The sterilization process was successful if no colony was found on agar plate after inoculation.

Total 1 g fresh plant was cut up and ground with 10 ml sterile deionized water in a sterile mortar. The grinding suspension (100 μl) was spread onto MSM agar plate containing 100 mg L^−1^ BSM and then cultured in an incubator at 37°C for 3 d. Colonies with clear zones were selected as potential BSM-degrading endophytic microbes.

### Identification of the endophytic bacterium

The strain CD3 with the highest BSM degradation rate from the above screening experiment was selected for further analysis. Colony growth and morphology were examined on LB agar plate, following incubation at 37°C for 3 d. Cell morphology, motility and Gram-reaction were observed under light microscope and scanning electron microscope. The basic physiological and biochemical characteristics of this endophyte were performed using traditional methods in Bergey’s manual. The 16S rDNA of strain CD3 was extracted and purified by commercial DNA extraction kit (Qiagen, German), and amplified by PCR with bacteria universal primers of 27F (5′-AGAGTTTGATCCTGGCTCAG-3′) and 1492R (5′-GGTTACCTTGTTACGACTT-3′). The PCR amplification process was as follows: denaturation at 95°C for 5 min, 40 cycles with each consisting of 95°C for 30 s, 55°C for 30 s and 72°C for 1 min, and a final extension at 72°C for 10 min. The PCR products were verified and purified by agarose gel electrophoresis. The purified PCR product was sequenced in the Shanghai Invitrogen Biological Technology Co., Ltd. and aligned with the published sequences in GenBank using BLAST program. An unrooted phylogenetic tree was constructed using the Neighbor-Joining method with bootstrap values estimated from 1,000 replicated analyses using MEGA software.

### Degradation experiments

#### Optimization of the BSM-degrading conditions

To study the effect of additional carbon sources on BSM degradation by strain CD3, 10 g L^−1^ glucose, 10 g L^−1^ peptone, and 5 g L^−1^ yeast powder were added into MSM with 50 mg L^−1^ BSM, respectively. The inoculated solutions in 250-ml Erlenmeyer flasks (in triplicate) were incubated under the suitable conditions for 7 d. Non-inoculated samples were kept as controls. Sample using BSM as the sole carbon source for culture was also used to compare the efficiency of BSM degradation.

The important parameters selected for the optimal degrading conditions were temperature, pH and the initial concentration of BSM, which could significantly influence the biodegradation of BSM by strain CD3. Single-factor test was designed under different conditions including incubation temperature (20–40°C), pH (5–9) and the initial concentration of BSM (12.5–400 mg L^−1^) in MMG. All samples in triplicate were incubated at 37°C for 7 d in a rotary shaker (180 rpm).

#### Degradation of BSM and other sulfonylureas by strain CD3

Degradation experiments were studied under the optimal conditions in MMG with 50 mg L^−1^ BSM, nicosulfuron, halosulfuron methyl, pyrazosulfuron, and ethoxysulfuron, respectively. The strain CD3 was pre-cultured in LB medium to exponential phase, inoculated into MMG and incubated for 7 d at 37°C with shaking at 180 rpm. Non-inoculated media with the same concentration of sulfonylurea herbicides were used as controls. At different time intervals (0 h, 6 h, 12 h, 1 d, 3 d, 5 d, and 7 d), samples of the culture media were withdrawn for the removal determination. The growth of strain CD3 was recorded by measuring the optical density (OD) value at 600 nm using ultraviolet–visible spectrophotometer (Evolution 220, Thermo Fisher Scientific, United States) during the degradation of BSM.

#### Determination of BSM and other sulfonylureas

The herbicide residues were detected by a Waters e2695 high-performance liquid chromatograph (HPLC) equipped with UV detector and an XDB C_18_ column (250 × 4.6 mm, 5 μm). The mobile phase was acetonitrile: water containing 0.05% acetic acid (v/v = 55/45) with a flow rate of 1 ml min^−1^. The detection wavelength was 235 nm and the injection volume was 10 μl with column temperature at 35°C. The degradation rate was calculated by the following equation:


$$\eta \left(\%\right)=\frac{C_{0}-C_{t}}{C_{0}}\times 100$$

where *C*_0_ (mg L^−1^) is the initial concentration of BSM, *C_t_* (mg L^−1^) is the residual concentration of BSM at time *t*.

### Degradation of BSM by crude enzymes

#### Extraction of crude enzymes

For the preparation of crude enzymes, strain CD3 cultivated for 16 h in LB medium was exposed to BSM with a concentration of 50 mg L^−1^ in MSM for 2 d at 37°C and 180 rpm for enzyme induction. Then the cells and supernatant were collected by centrifugation at 3380 x*g* and 4°C for 10 min.

After the supernatant was centrifugated at 9390 x*g* and 4°C for 10 min, ammonium sulfate was added to saturation, and the extracellular crude enzyme precipitate was salted out at 4°C for 16 h. The precipitate was washed twice with phosphate buffer solution (PBS, pH = 7), resuspended in 15 ml PBS and stored at −20°C for further study.

The obtained cells were washed three times with PBS, harvested by centrifugation (9390 x*g*, 10 min) at 4°C and resuspended in 15 ml PBS. The cells were sonicated for 8 min at 150 W and centrifuged at 9390 x*g* for 10 min at 4°C. The supernatant was intracellular enzyme and stored at −20°C. The cell debris containing membrane-bound enzyme was resuspended in 15 ml PBS and stored at −20°C.

#### Degradation of BSM by crude enzymes

The activities of different crude enzymes to BSM were determined by analyzing the residual amount of BSM in MSM. One unit of enzyme activity was defined as the amount of enzyme that catalyzed 1 μmol BSM per minute at 37°C. Crude enzymes were warmed at 37°C and then 0.5 ml crude enzymes were added into 2.5 ml MSM containing 50 mg L^−1^ BSM, respectively. After incubation for 2 h at 37°C, the samples were collected for the determination of BSM. Each treatment was carried out in triplicate, and the controls without the added crude enzymes were performed under the same conditions.

#### Optimal conditions of extracellular crude enzyme for degrading BSM

To optimize the incubation time, extracellular crude enzyme solution (0.5 ml) was added into 2.5 ml preheated MSM containing 50 mg L^−1^ BSM at 37°C. BSM concentration was detected after incubated for 0.5, 1, 2, 3 h, respectively.

To test temperature effect, 2.5 ml MSM containing 50 mg L^−1^ BSM was adjusted to the temperatures of 25, 30, 37, 42, 47, and 52°C, respectively. Extracellular crude enzyme solution (0.5 ml) was added and incubated for 2 h. BSM concentration was detected using the method described above.

Further, a series of 2.5 ml MSM containing 50 mg L^−1^ BSM with different pH values of 3, 4, 5, 6, 7, 8 were heated to 37°C, and 0.5 ml extracellular crude enzyme solution was added. After incubated for 2 h at 37°C, the concentration of BSM was detected.

In the above experiments, each treatment was carried out in triplicate, and the controls without the added extracellular crude enzyme were performed under the same conditions.

### Statistical analysis

The data were calculated using Excel 2019 and then analyzed with the SPSS 22.0 software and Origin 9.1 software. The statistical significance of differences was performed with the one-way analysis of variance (ANOVA) by Tukey’s test. Differences were considered significant at *p* values <0.05.

### Determination and identification of the degradation products of BSM

The degradation products of BSM were detected by triple quadrupole liquid chromatograph-mass spectrometer (LC–MS) with an electrospray ionization (ESI) source (Thermo Fisher Scientific, United States). An analytical column (Hypersil GOLD C_18_, 1.9 μm, 2.1 mm × 100 mm) was used with the column temperature at 35°C and the constant flow rate of 0.3 ml min^−1^. The ESI–MS condition was as follows: the spray voltage was set to 3,500 V in positive polarity mode, nitrogen was used as the sheath gas with the pressure of 40 psi, and the capillary temperature was set at 320°C. Full scans were obtained by scanning from m/z 80 to 250.

### Genome sequencing, assembly, and annotation

Genomic DNA was extracted from strain CD3 with the SDS method quantified by Qubit 3.0 Fluorometer (Thermo Scientific, United States), and then sequenced by using an Illumina HiSeq 2000 platform (San Diego, CA, United States) with a whole-genome shotgun (WGS) strategy at Beijing Genomic Institute (BGI, Shenzhen, China). The raw data were filtered, and then assembled by using SOAP denovo version 2.04, SSPACE version 2.0, Gap-Filler version 1.10, and BWA version 0.7.4, respectively. Genomic database of strain CD3 was submitted and deposited in NCBI (accession number: CP019686). The genome annotation was performed using the NCBI Prokaryotic Genomes Automatic Annotation Pipeline (PGAP).

## Results and discussion

### Isolation and identification of strain CD3

Total 13 endophyte strains named as CD1–CD13 were isolated and purified from barnyard grass. Among the 13 strains, strain CD3 had the highest degradation ability with more than 90% degradation rate of BSM within 3 d. Thus, further researches were focused on strain CD3.

**Figure 1:**
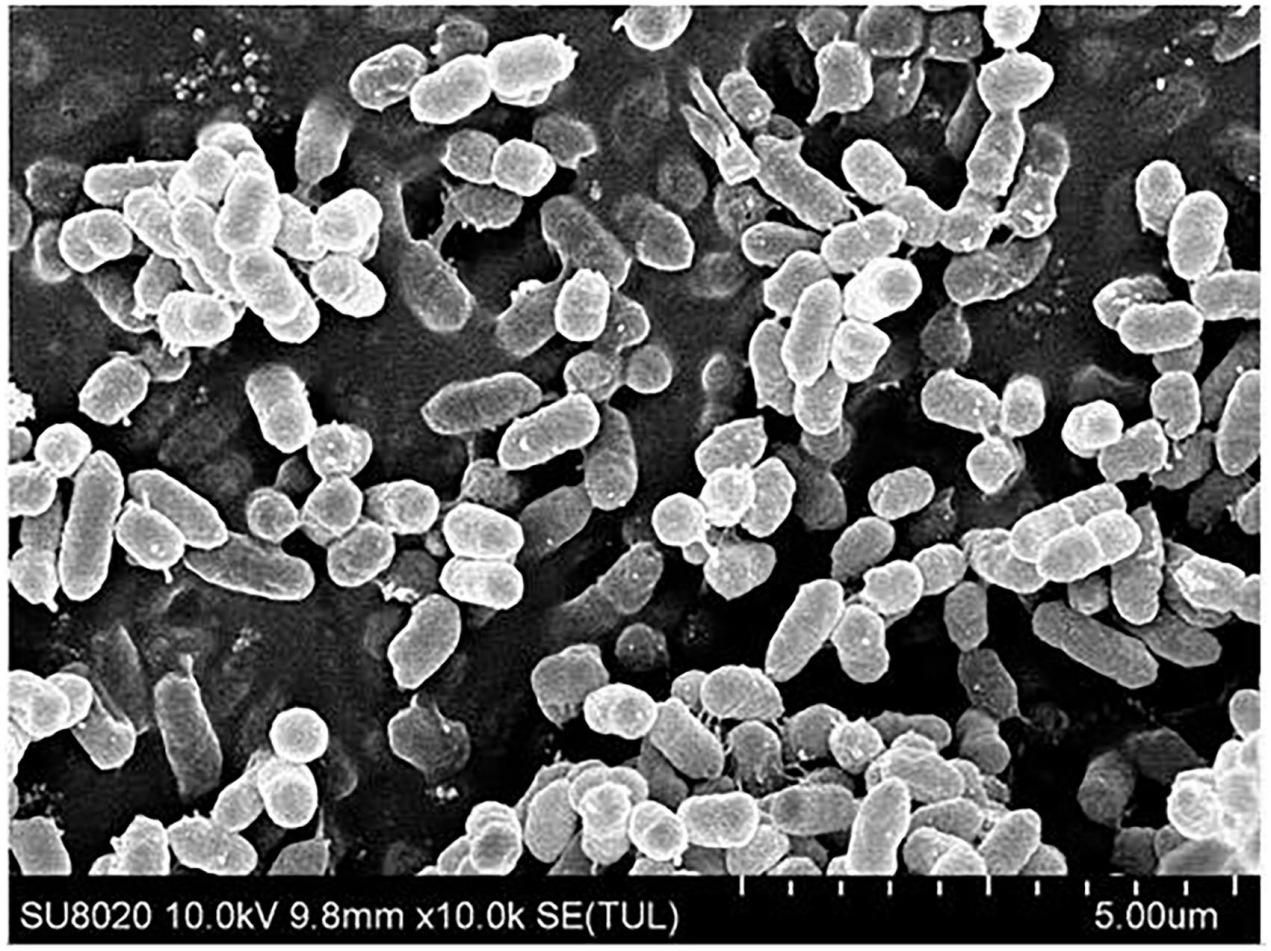
The cell morphology of strain CD3 under scanning electron microscope.

The morphological characteristic of strain CD3 was studied in this experiment. The colony of strain CD3 had characteristic of swarming growth when grown on LB agar plate for 1 d. The cell morphology of strain CD3 under scanning electron microscope illustrated in Figure 1 was short rod-like with the length in the range of 0.6–1.5 μm and the average width of 0.5 μm, which occurred singly or in short chains. This strain CD3 was an obligate aerobe (oxidase- and catalase-positive). It utilized glucose, but not galactose.

**Figure 2:**
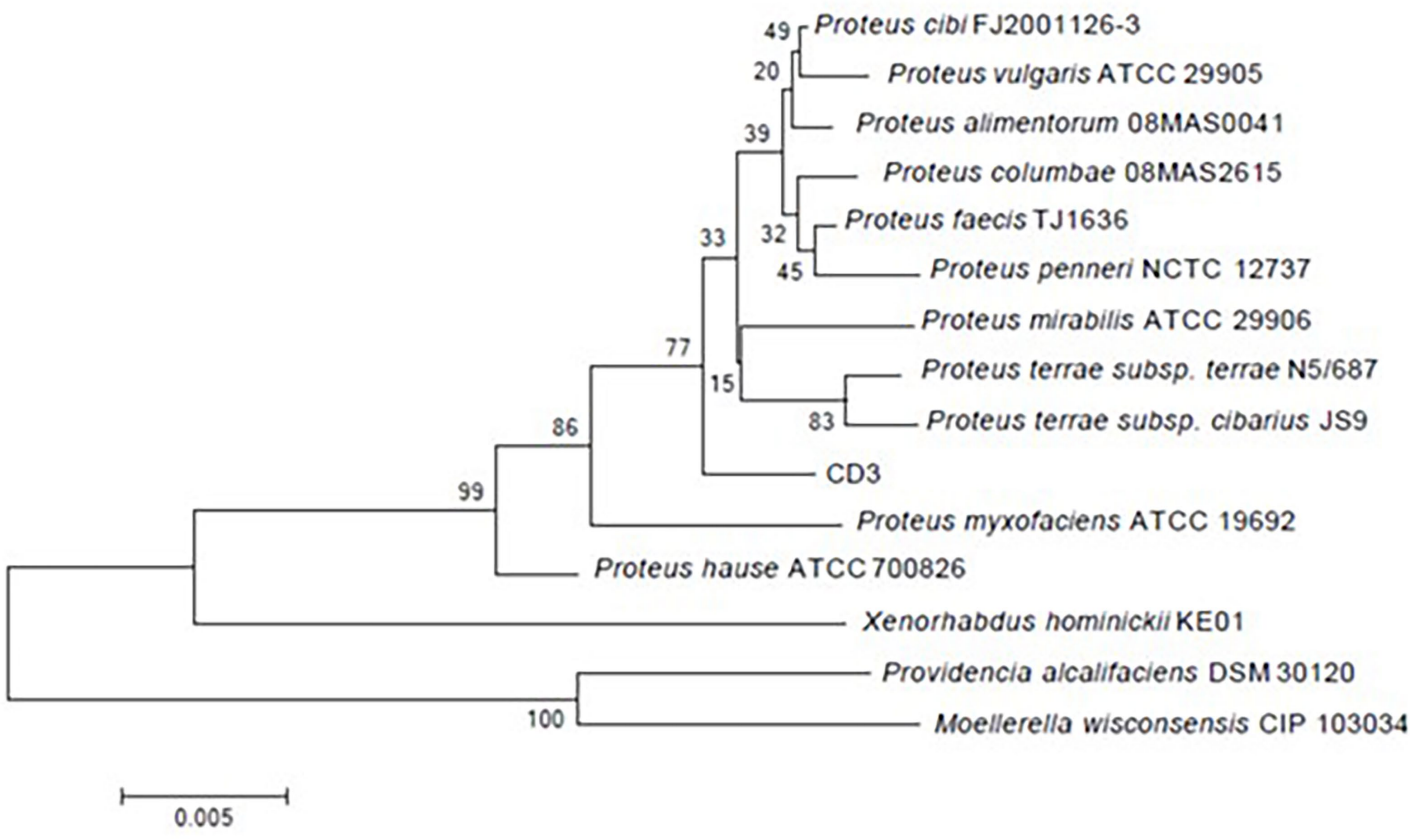
Phylogenetic tree constructed on the basis of 16S rRNA gene sequence of strain CD3 and other related species retrieved from the GenBank database according to neighbor joining. Bootstrap values obtained with 1,000 resamplings were indicated as percentages at all branches.

The amplification fragment of 16S rDNA (1,504 bp) was sequenced and deposited in GenBank under accession number ON376828. 16S rDNA alignment showed that strain CD3 had a high similarity with *P. alimentorum* 08MAS0041 (99.57%), *P. cibi* FJ2001126-3 (99.51%), *P. faecis* TJ1636 (99.28%), *P. vulgaris* ATCC 29905 (99.25%), *P. columbae* 08MAS2615 (99.13.0%), *P. mirabilis* 29,906 (99.04%), and *P. hauseri* ATCC 700826 (98.91%). Phylogenetic analysis (Figure 2) based on the 16S rDNA gene sequence revealed that strain CD3 belonged to the *Proteus* sp.

As stated above, strain CD3 was identified as a *Proteus* sp. strain according to the results of phenotypic features, physiological and biochemical characteristics, and 16S rDNA sequence analysis.

So far, various studies showed that many bacteria were able to degrade BSM, including *Brevibacterium* sp. (Zhu et al., 2005; Luo et al., 2008), *Ochrobactrum* sp. (Lü et al., 2008), *Bacillus megaterium* (Lin et al., 2010), *Rhodococcus* sp. (Xiong et al., 2011), *Methylopila* sp. (Duc and Oanh, 2020), *Edaphocola flava* (Hu et al., 2020), and *Hansschlegelia zhihuaiae* (Zhang et al., 2021), etc. However, BSM-degrading endophytic bacterium was not reported. This study was the first report that an endophytic bacterium degraded BSM, expanding the application range of endophytic bacteria in the field of environmental pollution remediation.

### Optimization of the BSM-degrading conditions

**Figure 3:**
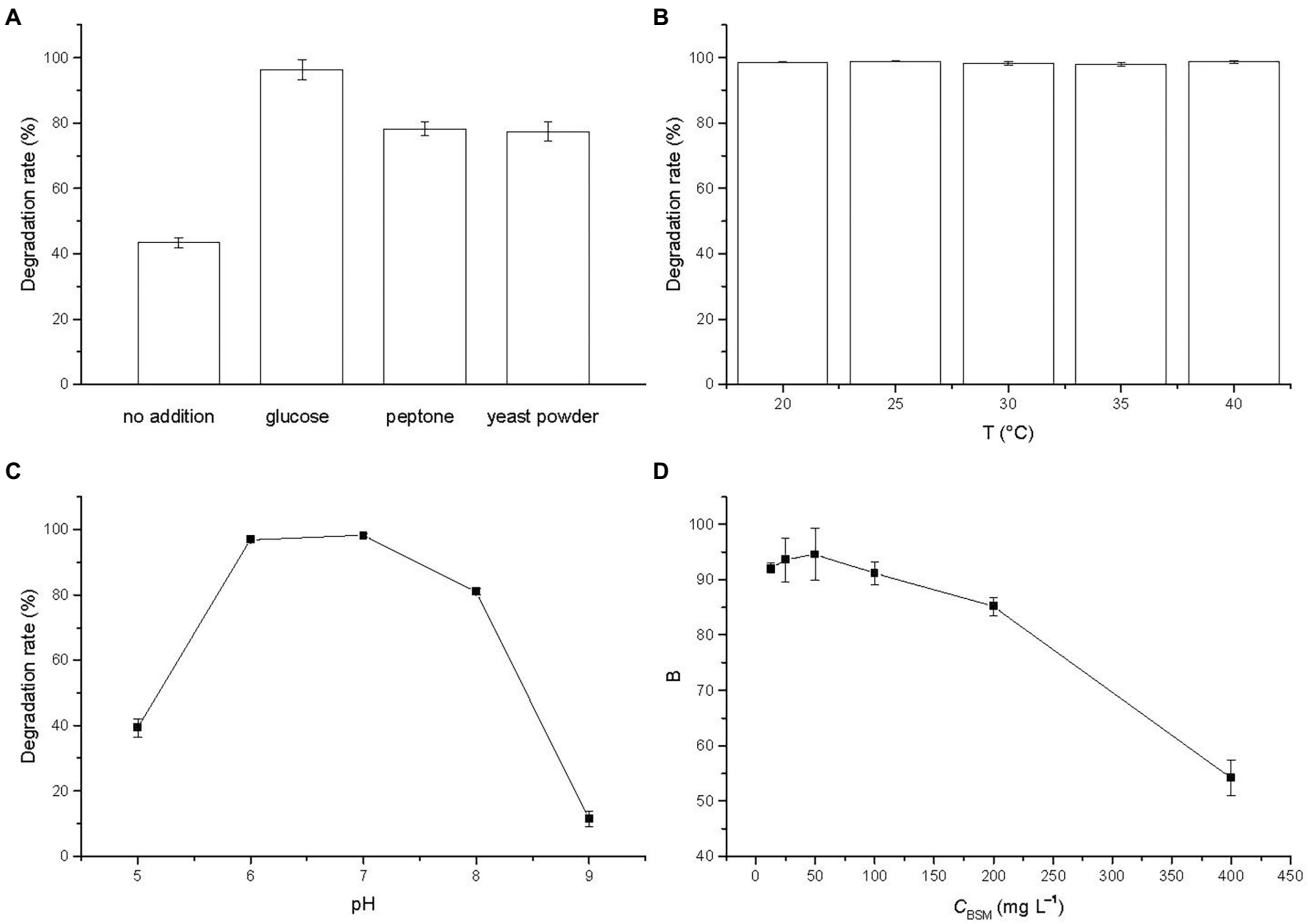
Effects of **(A)** cosubstrates (glucose 10 g L^−1^, peptone 10 g L^−1^, yeast powder 5 g L^−1^), **(B)** temperature, **(C)** pH, and **(D)** initial BSM concentration on the degradation of BSM by strain CD3. Error bars represent standard deviation (n = 3).

The degradation of BSM in MSM supplied with different carbon sources was studied and the result was illustrated in Figure 3A. The degradation rates were 43.42% and from 77.43 to 96.31% without and with the addition of carbon sources, respectively. The results revealed that BSM biodegradation in the presence of different carbon sources presented significantly higher values than the one that used BSM as a single carbon source, which suggested that BSM degradation by strain CD3 underwent a co-metabolic process, a very universal phenomenon in the degradation of pollutants (Luo et al., 2008; Li et al., 2022). The addition of other carbon sources might relieve the inhibition effect of BSM on strain growth by supplying nutrition and stimulated the degradation performances of strain CD3 (Luo et al., 2008; Duc and Oanh, 2020). The degradation rate reached the maximum of 96.31% in the presence of glucose. Thus, glucose was chosen as the additional carbon source supplied to MSM.

Temperature was an important factor on the biodegradation of organic pollutant. The degradation rate of BSM by strain CD3 at the temperature from 20 to 40°C was investigated and the result was illustrated in Figure 3B. The degradation rates at 20, 25, 30, 35, and 40°C were 98.61, 98.94, 98.23, 97.94 and 98.76% after incubation with strain CD3 for 3 d, respectively. The results showed that the degradation rates were fairly stable in the range of 20–40°C and presented a value above 97%, which illustrated that strain CD3 had an obvious adaptability to the temperature. A similar study was previously reported that CarE presented a fairly stable activity above 90% to the degradation of chlorimuron-ethyl from 15 to 40°C (Zang et al., 2020). Such efficient degradation at relatively wide temperature range promoted us to further investigate the degradation of BSM by crude enzymes. Considering the growth of strain CD3, 37°C was chosen in the degradation experiments.

The effect of pH values on the degradation of BSM was investigated after incubation with strain CD3 for 5 d and the result was shown in Figure 3C. The degradation rate of BSM initially increased with the increase of pH value. When pH was 7, the degradation rate reached the maximum value of 98.15%. Then, the degradation rate decreased with the further increase of pH value. The degradation rate at pH 9 was only 11.50%. The results indicated that the favorable pH range for BSM degradation was 6–8 with the optimal pH of 7.

To evaluate the effect of BSM concentration on the degradation, initial BSM concentrations were varied from 12.5 to 400 mg L^−1^ under the optimal conditions. Observed from Figure 3D, the degradation rates of BSM were above 90% in the initial concentration range of 12.5–100 mg L^−1^ within 7 d, and then were inhibited but remained a relatively high value of 85.22% at the initial concentration of 200 mg L^−1^. The result showed that BSM could be effectively degraded when the initial concentration was below 200 mg L^−1^, which illustrated that strain CD3 might be more valuable in the degradation of BSM. However, the inhibition was extended at higher BSM concentrations, with only 54.24% degradation achieved at the initial concentrations of 400 mg L^−1^. It was possible that the growth of strain CD3 or activity of degrading enzymes from strain CD3 had been inhibited with the increase of BSM concentration (Luo et al., 2008; Xiong et al., 2011). When the initial concentration was 50 mg L^−1^, the highest degradation rate was achieved after incubated for 7 d. Thus, 50 mg L^−1^ was chosen as the initial concentration of BSM in the further research.

### Degradation of BSM and other sulfonylureas by strain CD3

**Figure 4:**
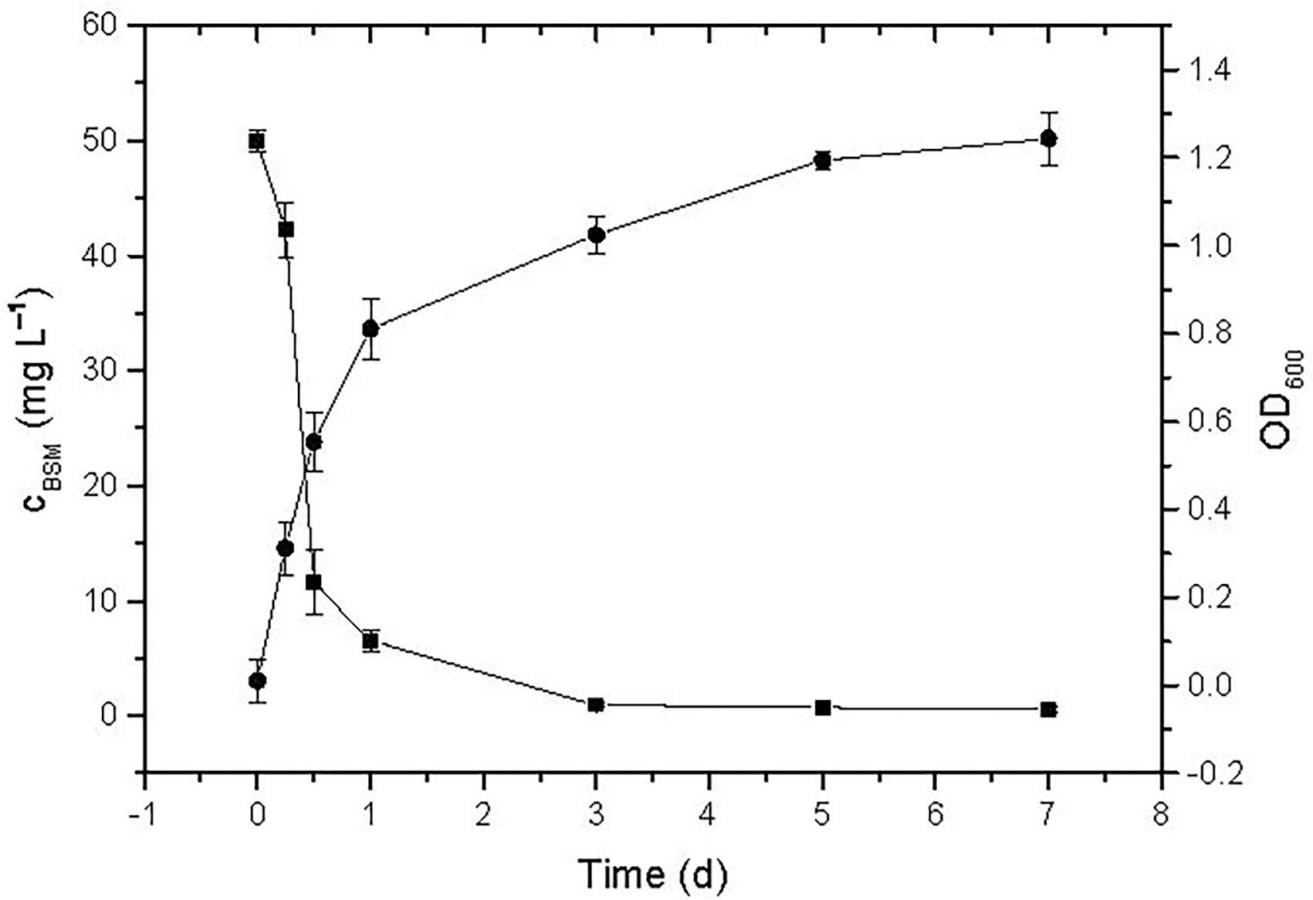
BSM residue (▪) and concomitant growth of strain CD3 (•) in MMG with 50 mg L^−1^ BSM under the optimal condition.

The BSM residue and growth pattern of strain CD3 were measured in amending MMG with 50 mg L^−1^ BSM. Observed from Figure 4, the concentration of BSM residue decreased rapidly in the first day, then slowly changed in the next 2 d, finally presented a slight decrease at the last 2 d. The change of BSM residue indicated that BSM was rapidly degraded by strain CD3 and more than 97% of the initial concentration was degraded within 3 d. Meanwhile, no significant change in BSM concentration was observed in non-inoculated cultures (data not shown). BSM degradation was associated with a concomitant increase in cell density from 0.01 to 1.24. The decrease in BSM residue associated with the increase of OD_600_ indicated that strain CD3 could utilize BSM for their growth.

**Figure 5:**
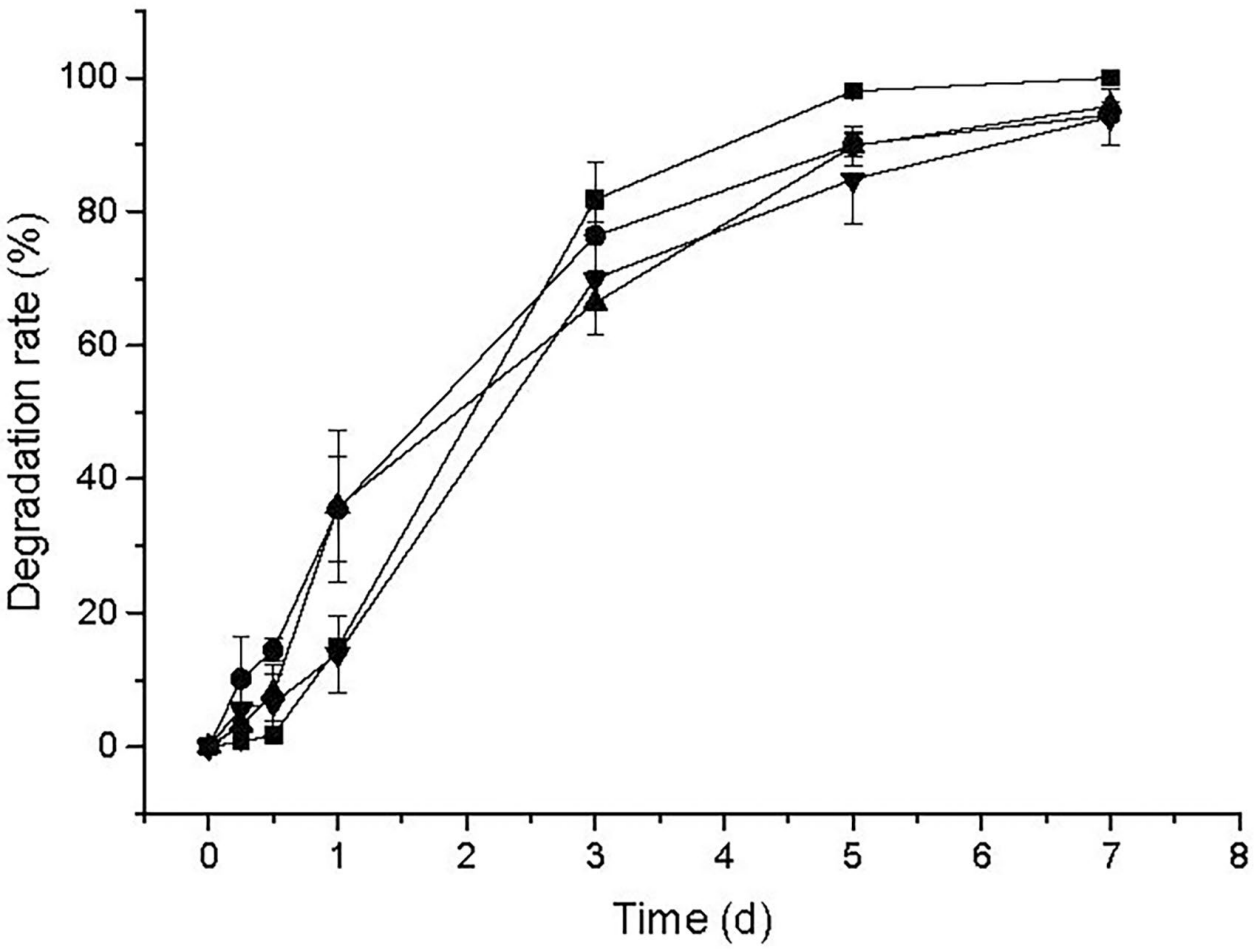
Degradation of other sulfonylurea herbicides including nicosulfuron (▪), halosulfuron methyl (•), pyrazosulfuron (▴), and ethoxysulfuron (▾) by strain CD3.

To test the degradation ability of strain CD3, other sulfonylurea herbicides were measured in this study and the degradation results were illustrated in Figure 5. Nicosulfuron, halosulfuron methyl, pyrazosulfuron, and ethoxysulfuron were degraded 98.01, 89.98, 89.84, and 84.84% after 5 d of incubation in MMG at the initial concentration of 50 mg L^−1^, respectively. It was noteworthy that the degradation rate of nicosulfuron reached 100% after incubation for 7 d, suggesting that strain CD3 might have even higher degrading ability to nicosulfuron than BSM. These results showed that strain CD3 had a broad degradation spectrum of various sulfonylurea herbicides. Strain CD3 may possess potential to be used in the bioremediation of sulfonylurea herbicide-contaminated environments with relatively high efficiency.

In previous reports, strains S113, LW3, XJ-412-1, LAM-WHM-ZC, and CF57 could also degrade BSM and other different sulfonylurea herbicides with the different degradation rates (Huang et al., 2007; Ma et al., 2009; Lu et al., 2011; Zhou et al., 2017; Zhang S. S. et al., 2020). In addition, strains ZWS11 and ZWS16 were capable of degrading some other sulfonylurea herbicides, however, BSM could not be degraded under the same conditions (Zhao et al., 2015a, 2015b). These differences in the substrate ranges and degradation rates might imply that the relevant degradative pathways or at least the initial reactions were different in these strains.

### Degradation of BSM by crude enzymes

**Figure 6:**
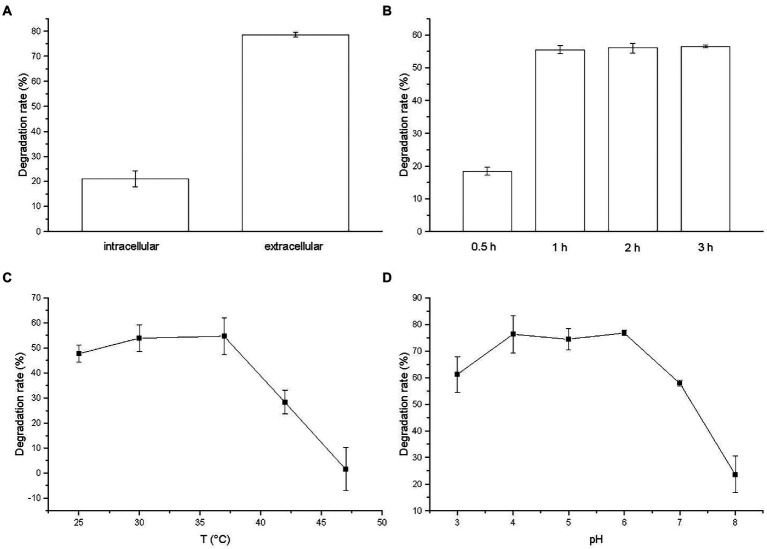
**(A)** Relative activities of crude enzymes to BSM; Effects of **(B)** degradation time, **(C)** temperature, and **(D)** pH on the degradation of BSM by extracellular crude enzyme.

The degradation of BSM by membrane-bound enzyme, intracellular enzyme and extracellular enzyme was observed and the result was illustrated in Figure 6A. No activity was presented by membrane-bound enzyme in cell debris (data not shown). Extracellular enzyme yielded a degradation rate of 78.61%, which was higher than intracellular enzyme with a degradation rate of 21.14%. The result indicated that extracellular enzyme played a critical role on the BSM degradation by strain CD3, which was consistent with the localization of degradation enzyme in sulfonylurea herbicide-degrading bacteria (Kang et al., 2014; Zhang S. S. et al., 2020).

The effect of incubation time on the activity of BSM-degrading enzyme was investigated and the result was illustrated in Figure 6B. The degradation time could obviously affect the activity of extracellular enzyme. With the increase of degradation time, the degradation rate of BSM by extracellular enzyme increased. The BSM degradation occurred rapidly and the degradation rate reached 55.56% after incubated for 1 h. The degradation rate got a slight increase when the degradation time increased further to 3 h. Thus, we chose 2 h as the degradation time in the following study.

The percentage of BSM degradation by the extracellular crude enzyme increased initially with temperature, and then reached the maximum at 37°C with a degradation rate of 54.71%, finally decreased with increasing temperature (Figure 6C). The enzyme yielded a degradation rate of 28.37% at 42°C, but was almost completely inactivated at 47°C with a very low degradation rate of 1.57%. The result suggested that temperature might be related to the activity of enzyme for degrading BSM and high temperature greatly reduced the activity of BSM-degrading enzyme as the report of the nicosulfuron-degrading enzyme (Kang et al., 2012).

Observed from Figure 6D, the optimal pH value of the degradation activity by extracellular enzyme was 6. Meanwhile, the enzyme activity for degrading BSM was stable at the pH values ranging from 3 to 7, retaining more than 57% of the degradation rate, which suggested that extracellular enzyme from strain CD3 had an effective broad pH range for BSM degradation. The degradation rate decreased rapidly when pH increased to 8. The results indicated that BSM degradation by extracellular enzyme was favored by the acidic solution, whereas alkaline condition inhibited the degradation.

As suggested above, extracellular crude enzyme might be responsible for BSM degradation by strain CD3 and the maximum enzyme activity was obtained at 37°C and pH 6.

### Degradation products and pathways of BSM

**Figure 7:**
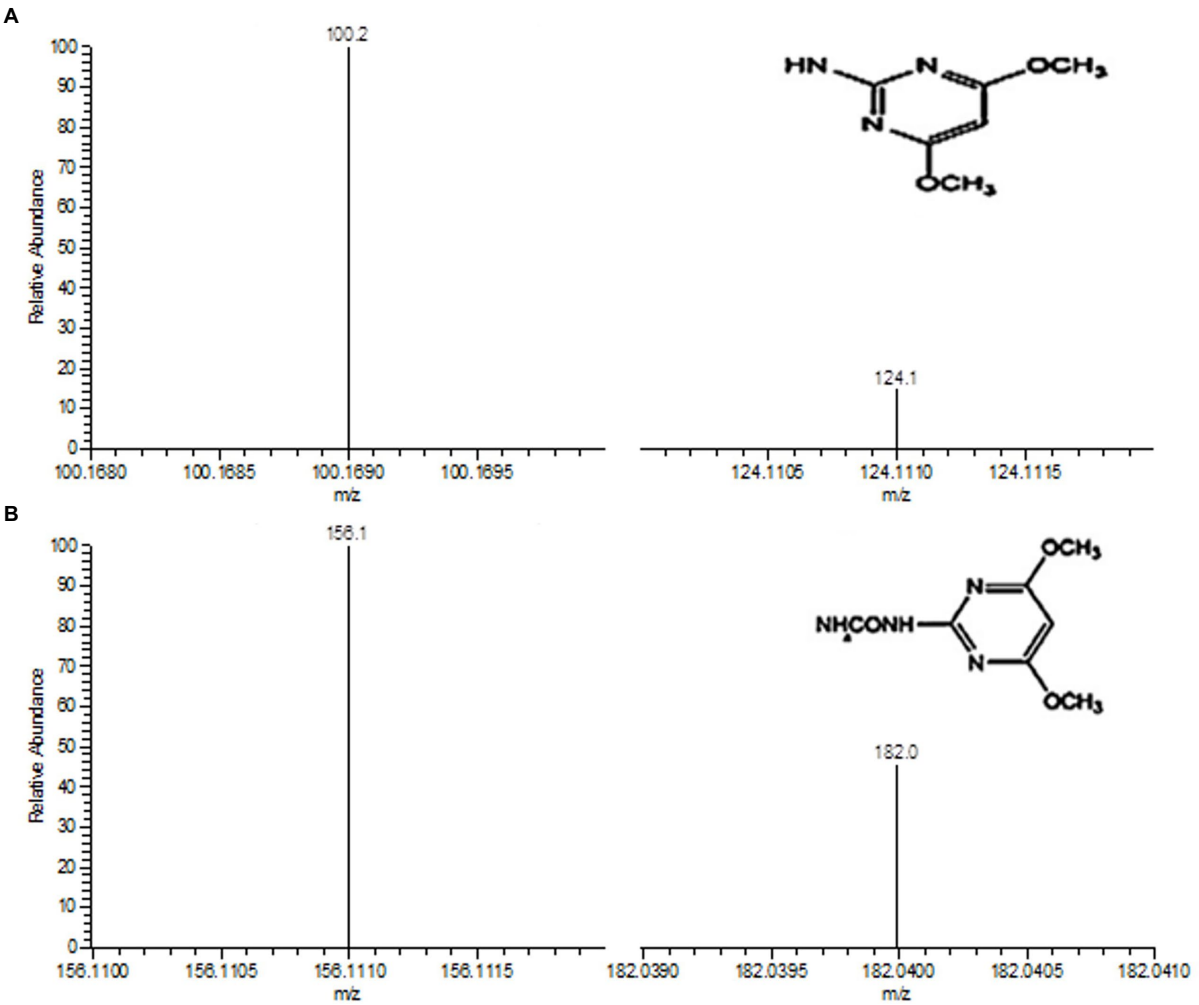
Determination of degradation metabolites 1 **(A)** and 2 **(B)** by LC–MS. The insets in A and B are the chemical structures of metabolites 1 and 2, respectively.

The degradation products of BSM after incubation for 3 d were extracted and identified by LC–MS. Two metabolites were observed with retention times (RTs) of 2.34 and 3.42 min, respectively. The major metabolite 1 with fragments at m/z 100.2 and 124.1 occurred in the mass spectrum (Figure 7A) and was identified as 2-amino-4,6-dimethoxypyrimidine (ADMP, C_6_H_8_N_3_O_2_), which was a common metabolite produced in the degradation of sulfonylurea herbicides from the cleavage of the C–N bond in the sulfonylurea bridge (Perreau et al., 2007; Peng et al., 2012; Carles et al., 2017, 2018; Duc and Oanh, 2020). In addition, a minor metabolite 2 of BSM degradation was identified with the mass ion fragments at m/z 156.1 and 182.0 (Figure 7B). Metabolite 2 was identified as 1-(4,6-dimethoxypyrimidin-2-yl) urea (DMPU, C_7_H_12_N_4_O_3_), which was derived from the cleavage of sulfonyl amide linkage and similar to that described in the previous reports (Peng et al., 2012; Zhu et al., 2018).

**Figure 8:**
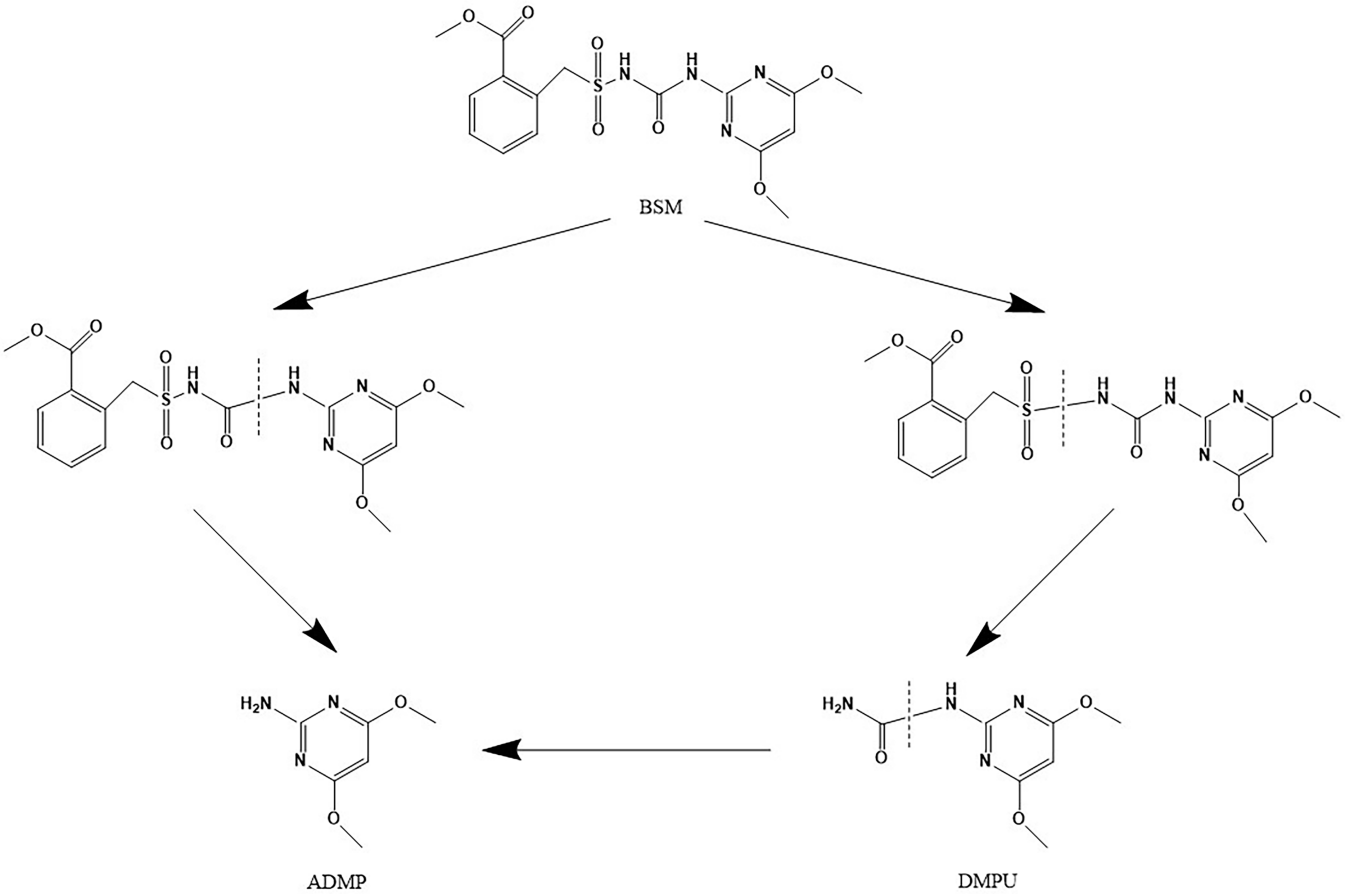
Proposed pathways for the degradation of BSM by strain CD3.

According to the results of LC–MS analysis and chemical properties of sulfonylurea herbicides as described in related reports (Perreau et al., 2007; Peng et al., 2012; Sharma et al., 2012; Carles et al., 2017, 2018; Zhu et al., 2018; Duc and Oanh, 2020), two metabolic pathways were proposed for BSM biodegradation by strain CD3 (Figure 8). A possible transformation pathway was the cleavage of the C–N bond in the sulfonylurea bridge to form metabolite 1 (ADMP), which was previously proposed as the predominant metabolic pathway of sulfonylurea herbicides (Peng et al., 2012; Zhu et al., 2018). The other degradation pathway was the cleavage of sulfonyl amide linkage to produce metabolite 2 (DMPU), then form metabolite 1 (ADMP) through the further urea hydrolysis (Sharma et al., 2012; Zhu et al., 2018).

### Genome of *Proteus* sp. strain CD3

**Figure 9:**
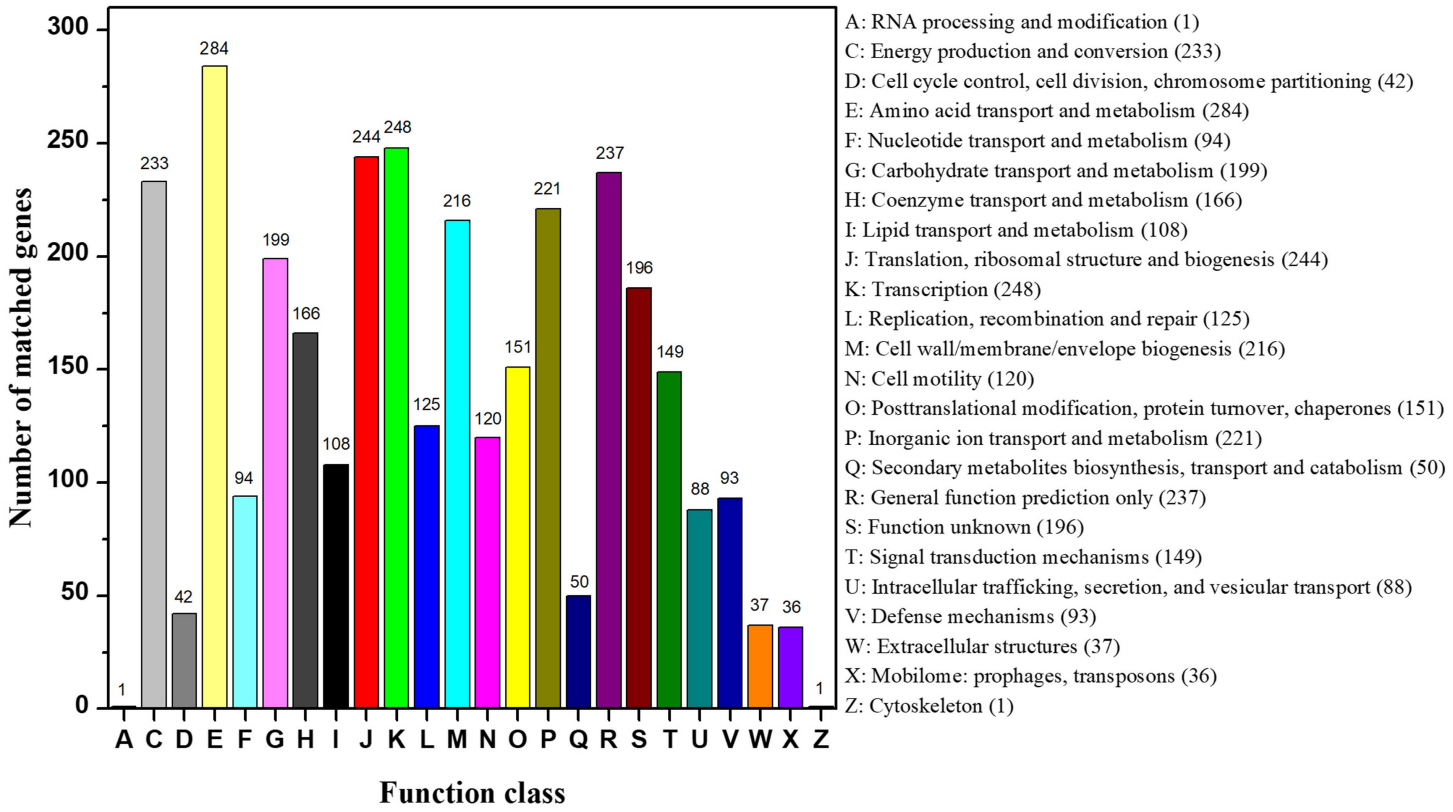
COG function classification of CD3 strain.

The complete genome of strain CD3 had a single chromosome of 4,056,724 bp with a G + C content of 39.23%. The genome contained 3,638 predicted protein coding genes (CDS) with an average size of 951 bp, giving a coding intensity of 85.28%. Analysis revealed 85 tRNA genes, and 22 rRNA (5 s RNA genes, 16 s RNA genes, and 23 s RNA genes) in the genome. Average nucleotide identity (ANI) analysis revealed that *Proteus* sp. CD3 was phylogenetically related to *P. columbae* 08MAS2615 (94.07%), *P. alimentorum* 08MAS0041 (93.47%), *P. cibi* FJ2001126-3 (92.65%), and *P. faecis* TJ1636 (91.92%). Digital DNA/DNA hybridization analysis showed that *Proteus* sp. CD3 was the closest to *P. columbae* 08MAS2615 (82.0%), and had similarity with *P. cibi* FJ2001126-3 (80.5%), *P. alimentorum* 08MAS0041 (78.5%), *P. faecis* TJ1636 (77.0%), *P. hauseri* ATCC 700826 (66.3%), and *P. mirabilis* ATCC 29906 (53.1%) by using the TYGS web page. Among the 3,638 CDS, 2951 could be assigned to 24 different categories of clusters of orthologous groups (COGs). These results clearly suggested the organism’s efficient lipid, carbohydrate, and amino acid transport and metabolism for energy (Figure 9).

In the present study, strain CD3 was found to be able to convert BSM to ADMP or DMPU. Total 28 gene sequences annotated with KEGG pathways were clustered into xenobiotics biodegradation and metabolism, involved in hydrolases, lyases, isomerases, transferases, oxidoreductases. Currently, few studies focus on enzymes and their coding genes which involved degradation of sulfonylurea herbicides. In previous reports, sulfonylurea herbicides were mainly degraded through esterases or hydrolases in microorganism (Yu et al., 2022). The hydrolases encoded by P450SU1 and P450SU2 could hydrolyze and oxidize the urea bridge of sulfonylurea herbicide (O’Keefe and Harder, 1991). An esterase gene *sulE*, cloned from *H. zhihuaiae* S113, was involved in the de-esterification of chlorimuron-ethyl (Hang et al., 2012). Moreover, the esterase E3 identified from *Oceanisphaera psychrotolerans* can break the sulfonylurea bridge of nicosulfuron (Zhou et al., 2017). Recently, a carboxylesterase gene, *carE*, was identified from *R. erythropolis* D310-1,which degraded chlorimuron-ethyl through the de-esterification pathway (Zang et al., 2020). Finally, CarE function was certified using gene knockout, complementation, expression, and purification of recombinant. The gene *Kj-gst* encoding GST was cloned from *Klebsiella jilinsis* 2 N3 catalyzing the degradation of chlorimuron-ethyl. GST function involved in the degradation of chlorimuron-ethyl was confirmed using gene knockout and complementation, prokaryotic expression, and point mutation analyses (Zhang C. F. et al., 2020). Yu et al. (2022) predicted that nine enzymes (AtzF, AtzD, CysJ, SulE, GST, CopA, CrtD, CrtC, and Limb) from *Chenggangzhangella methanolivorans* strain CHL1 were involved in the degradation of chlorimuron-ethyl through three pathways, including a novel pyrimidine-ring-opening pathway. The gene knock-out and complementation results confirmed that three genes (*atzF, atzD*, and *cysJ*) were involved in chlorimuron-ethyl degradation by strain CHL1. The studies indicated microorganism with different genes had diversity metabolic mechanism to sulfonylurea herbicides. In this study, metabolic pathway of BSM were predicted to hydrolyze and oxidize the urea bridge. The genome of strain CD3 and metabolic pathway of BSM results indicated *sul*E, *carE* and *P450s* gene were involved in the biodegradation of BSM.

The *sul*E, *carE* and *P450s* gene sequences in the genome of strain CD3 showed relatively low amino acid similarities to those of previously reported enzymes. These results indicated novel degradation genes of BSM were present in strain CD3. The gene knock-out and complementation would be performed in future experiments. Cloning and expression of these putative esterases and hydrolases gene would be helpful for better understanding the molecular mechanism of BSM-degradation in genus *Proteus*.

## Conclusion

An entophytic bacterium strain CD3 was isolated from barnyard grass and identified as *Proteus* sp. This strain had a broad degradation spectrum of sulfonylurea herbicides including BSM, nicosulfuron, halosulfuron methyl, pyrazosulfuron, and ethoxysulfuron. The possible degradation pathways were the cleavage of the sulfonylurea bridge and the sulfonyl amide linkage. The genomic analysis of strain CD3 revealed the underlying mechanism and degradation pathways for BSM. The annotated functional roles of strain CD3 genes to detoxify the BSM needs to be verified in the future.

## Data availability statement

The datasets presented in this study can be found in online repositories. The names of the repository/repositories and accession number(s) can be found in the article/supplementary material.

## Author contributions

YW: conceptualization, methodology, and writing—original draft. XC: investigation and writing—original draft. HL: investigation. YM: resources. DZ: supervision. LD: supervision and writing—reviewing and editing. DJ: methodology. YW and XC contributed equally to this work. All authors contributed to the article and approved the submitted version.

## Funding

This work was supported by National Natural Science Foundation of China (no. 31660524).

## Conflict of interest

The authors declare that the research was conducted in the absence of any commercial or financial relationships that could be construed as a potential conflict of interest.

## Publisher’s note

All claims expressed in this article are solely those of the authors and do not necessarily represent those of their affiliated organizations, or those of the publisher, the editors and the reviewers. Any product that may be evaluated in this article, or claim that may be made by its manufacturer, is not guaranteed or endorsed by the publisher.
